# Rapidly progressive respiratory failure due to antisynthetase syndrome related interstitial lung disease

**DOI:** 10.1002/rcr2.1141

**Published:** 2023-04-12

**Authors:** Marwan Almubarek, Darryl P. Boy, Usha Lalla, Brian W. Allwood, Elvis M. Irusen, Coenraad F. N. Koegelenberg

**Affiliations:** ^1^ Division of Pulmonology, Department of Medicine Stellenbosch University & Tygerberg Hospital Cape Town South Africa

**Keywords:** anti‐Jo‐1 antibody, anti‐Ro52 antibody, antisynthetase syndrome, interstitial lung disease, intravenous immunoglobulin

## Abstract

A 65‐year‐old female was admitted with rapidly progressive respiratory failure requiring intubation and mechanical ventilation. She was considered to have an infective exacerbation of underlying interstitial lung disease (ILD). She improved on antibiotics, but the interstitial process progressed rapidly, and she could not be weaned. An antimyositis antibody panel yielded a strongly positive anti‐Jo‐1 and anti‐Ro 52. A diagnosis of antisynthetase syndrome (ASS) associated ILD, a very rare disease with high mortality, was made. She was managed with high‐dose corticosteroids and intravenous immunoglobulin therapy and was eventually liberated from mechanical ventilation. This case highlights the importance of considering ASS in an otherwise unexplained rapidly progressive ILD requiring mechanical ventilation.

## Graphical abstract

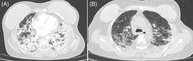

## INTRODUCTION

Antisynthetase syndrome (ASS) is a rare but probably underdiagnosed autoimmune disorder.^1^, ^2^ It is characterized by the presence of IgG anti‐aminoacyl RNA‐synthetase antibodies, with various clinical manifestations which may be present at different stages of the disease. These include autoimmune inflammatory polymyositis (PM), dermatomyositis (DM), interstitial lung disease (ILD), arthritis, fever, Raynaud's phenomenon and mechanic's hand.^1^, ^2^, ^3^

## CASE REPORT

A 65‐year‐old woman was referred with rapidly progressive respiratory failure, initially thought to be caused by community acquired pneumonia (CAP). She was intubated and mechanically ventilated at a district hospital and transferred to our intensive care unit (ICU).

Collateral history obtained from her son, however, suggested a 6‐month history of progressive dyspnoea and impaired exercise tolerance, with a one‐week history of coughing, fever, and rapid decline. Her past medical history included Hashimoto's thyroiditis. She also had a history of previous breast and previous cervical cancer, both treated with curative intent.

She was considered to have an infective exacerbation of underlying interstitial lung disease. In addition to signs of consolidation of the lower lobes, she was febrile and shocked on admission. There were no signs suggestive of systemic disease.

**FIGURE 1:**
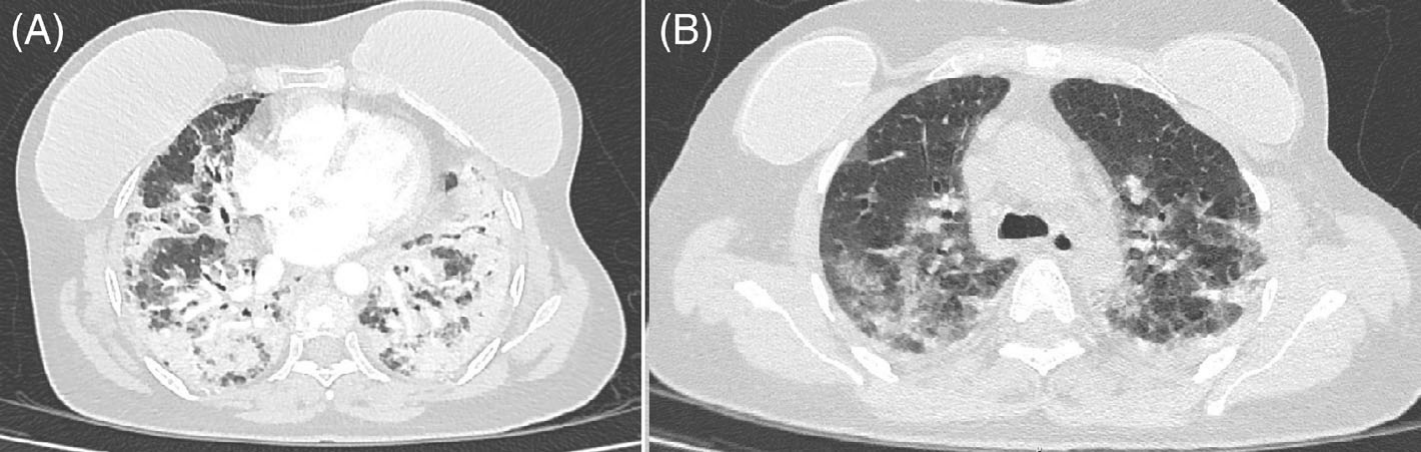
(A) A Computed tomography scan at presentation demonstrated confluent opacification with air‐ bronchograms consistent with pneumonic consolidation. (B) A higher slice showed patchy ground glass opacifications in both lung fields consistent with an interstitial process. Breast prostheses are visible on both slices

Her initial imaging was suggestive of consolidation, although some features suggestive of an underlying ILD were present as well (Figure 1). She had an elevated white cell count of 47.9 × 10^9^/L (normal 3.90–12.60) and C‐reactive protein (CRP) of 239 mg/L (normal <5 mg/L). Initial blood cultures were all negative, her tracheal aspirates were Gene Xpert negative, and her urine Legionella antigen test was negative.

She initially required significant ventilatory support with high positive end‐expiratory pressure (PEEP) and required high‐dose adrenalin (0.02 mcg/kg/min) to maintain adequate tissue perfusion. She was commenced on co‐amoxiclav and azithromycin at the district hospital but given the continuing rapid deterioration, resistant organisms were considered, and her antibiotics were escalated to empiric Meropenem.

The patient was liberated from vasopressor support and her ventilatory support was weaned within 5 days of admission. She had two failed attempts at extubation, on both occasions she was successfully extubated for 24 h, and subsequently became tachypnoeic and desaturated, requiring reintubation.

**FIGURE 2:**
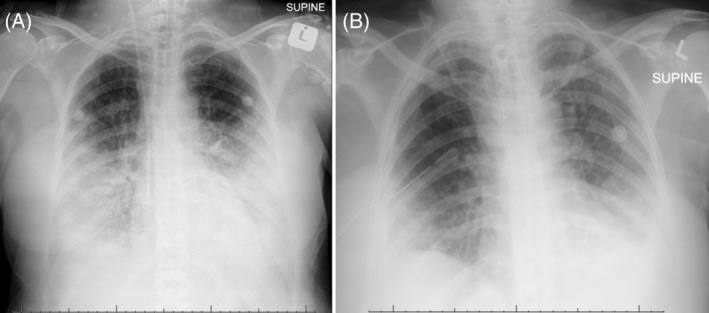
(A) The chest radiograph on admission illustrating bilateral lower zone pneumonic consolidation. (B) A chest radiograph following steroid therapy and later IV immunoglobulin therapy showing marked resolution (an iatrogenic pneumothorax was also present)

Her infective parameters improved (CRP to 16 mg/L), and imaging showed radiographic improvement of the consolidation, but worsening ground glass opacities and interstitial infiltrates (Figure 2).

Relevant special investigations revealed an elevated creatine kinase (CK) level of 1876 U/L (normal: 20–180 U/L), a negative antinuclear antibody, anti‐double stranded DNA antibody, anti‐SS‐A (Ro) antibody, anti‐SS‐B (La) antibody, and antineutrophil cytoplasmic antibodies. An antimyositis antibody panel showed a strongly positive anti‐Jo‐1 and anti‐Ro52.

A diagnosis of antisynthetase syndrome was made (ASS), and she was initially treated with high dose corticosteroids (hydrocortisone 100 mg 6‐hourly), followed by five daily infusions of IV immunoglobulin (IVIG) at a dosage of 400 gm/kg. She was once again weaned to minimal settings, and her CK dropped to 327 U/L.

Her ICU stay was complicated by several episodes of nosocomial sepsis (including two episodes of ventilator‐associated pneumonia (*Serratia marcescens* and *Stenotrophomonas maltophilia*)), candidaemia (*Candida albicans*) as well as an iatrogenic pneumothorax. She was eventually successfully weaned and liberated from the ventilator, discharged from ICU on oral prednisone (1 mg/Kg/day) to a high care setting and after almost 3 months after presentation, discharged home without the need for domiciliary oxygen. She is still maintained on oral prednisone with the view to review the management and potentially add a steroid‐sparing immunosuppressive agent, in all probability, Mycophenolate Mofetil.

## DISCUSSION

The classic triad of ILD, myositis, and arthritis accounts for up to 90% of ASS.^1^ However, recent evidence suggests that single‐organ involvement at presentation is far more common than originally described.^1^ ILD is the most frequent and often dominant organ involvement in Jo1 antibody‐associated antisynthetase syndrome, as seen in our case.^2^

The syndrome is generally considered to present in patients with an antisynthetase antibody plus one or two of the following features: ILD, inflammatory myopathy or inflammatory polyarthritis.^3^ Raynaud's phenomenon, mechanic's hands, and fever are other features that are less frequent clinical findings. ASS associated ILD often confers a poor prognosis and can be fatal despite aggressive treatment with corticosteroids.

The co‐occurrence of anti‐Jo1 and anti‐Ro52 antibodies in the antisynthetase syndrome has been related to more severe ILD and acute onset respiratory failure that is associated with a poor prognosis.^2^ At present, eight anti‐aminoacyl tRNA‐synthetase antibodies (anti‐ARS) have been identified,^1^, ^2^, ^3^, ^4^, ^5^ including anti‐Jo1 (most common), anti‐PL7, anti‐PL12, anti‐EJ, anti‐OJ, anti‐KS, anti‐ZO, and anti‐YRS/HA.^5^

There is a lack of evidence to guide treatment strategies in ASS‐ILD, and most suggested therapies are based on case reports and retrospective studies.^5^, ^6^ The proposed mechanisms of action of IVIG include alterations in the function of innate and adaptive immunity, as well changes in inflammatory cytokine production and gene expression.^7^ Its role in inhibiting autoantibodies and lack of immunosuppressive effect, make IVIG the preferred initial therapeutic agent in many centres.^5^ It can also be used as a potential salvage therapy in patients with active progressive ASS‐associated ILD who are not responding to corticosteroids. Furthermore, IVIG has be shown to have a steroid‐sparing effect in the management of ASS‐associated ILD.

According to a meta‐analysis in 2017, other available treatment options include corticosteroids, calcineurin inhibitors, azathioprine, cyclophosphamide, and Rituximab, with cyclophosphamide having the highest survival rate at 3 months (72.4%).^8^ Of note, no studies included in this meta‐analysis addressed the use of mycophenolate mofetil.

In conclusion, this case highlights the importance of considering ASS in an otherwise unexplained rapidly progressive ILD resulting in respiratory failure.

## AUTHOR CONTRIBUTIONS

All authors were involved in the clinical management of the patient. MA and CK wrote the manuscript, that was critically reviewed by all co‐authors.

## CONFLICT OF INTEREST STATEMENT

Coenraad FN Koegelenberg is an Editorial Board member of Respirology Case Reports and a co‐author of this article. They were excluded from all editorial decision‐making related to the acceptance of this article for publication. Coenraad FN Koegelenberg is an Associate Editor for Respirology Case Reports. The other authors have no conflict of interest to declare.

## ETHICS STATEMENT

The authors declare that appropriate written informed consent was obtained for the publication of this manuscript and accompanying images.

## Data Availability

Data available on request from the authors
